# Addressing distribution equity in spatial conservation prioritization for small-scale fisheries

**DOI:** 10.1371/journal.pone.0233339

**Published:** 2020-05-19

**Authors:** Alessia Kockel, Natalie C. Ban, Maycira Costa, Philip Dearden

**Affiliations:** 1 Geography Department, University of Victoria, Victoria, British Columbia, Canada; 2 School of Environmental Studies, University of Victoria, Victoria, British Columbia, Canada; Tanzania Fisheries Research Institute, UNITED REPUBLIC OF TANZANIA

## Abstract

Spatial conservation prioritization is used worldwide for designing marine protected areas (MPA) that achieve set conservation objectives with minimal impacts to marine users. People involved in small-scale fisheries (SSF) may incur negative and disproportionate impacts from implementing MPAs, yet limited available data often restricts their representation in MPA planning. Using a Philippines case study, we focus here on the systematic design of a MPA network that aims to minimize and distribute costs equitably for SSF whilst achieving representation targets for biodiversity conservation. The objectives of the study are to: (1) document a participatory mapping approach for collecting SSF data for prioritization using the local knowledge of fishers; and (2) examine how the completeness and resolution of SSF data may affect prioritization outputs in terms of biodiversity representation, spatial efficiency, and distribution equity. In the data-poor region, we conducted participatory mapping workshops with fishers in 79 communities to collect data on the spatial distribution patterns of different SSF fisheries and communities, and employed remote sensing techniques to define coastal habitats, which were targeted for inclusion in MPAs. The datasets were integrated within the decision-support tool Marxan with Zones to develop three scenarios. The SSF data incorporated in each scenario varied based on their completeness (considered all fishing methods or only dominant methods) and resolution (fishing methods itemized by community or municipality). All scenarios derived MPA plans that met representation targets with similar area coverage. The outputs, however, varied in terms of distribution equity, measured by the distribution of opportunity costs (loss of fishing grounds) across different fisheries and communities. Scenarios that did not include minority fisheries or variations between communities, led to inequitable costs. These results highlight the need to incorporate detailed data on SSF at appropriate resolutions, and how this can be achieved through participatory approaches.

## Introduction

The establishment of marine protected areas (MPAs) is a key global strategy in addressing ongoing declines in biodiversity and fisheries [1,2]. In the past two decades, treaties such as the Convention on Biological Diversity [3] and the Coral Triangle Initiative on Coral Reefs, Fisheries, and Food Security [4] have driven signatory governments to expand the coverage and representation (protection of representative samples of biodiversity) of their MPA system [2,5]. Further progress is needed to actually meet national commitments to treaties in order to address biodiversity loss [1,2,5,6]. However, continuing to increase the coverage and representation of MPAs will inevitably lead to greater spatial restriction on human access to marine resources [7–9]. People reliant on small-scale fisheries (SSF) are particularly vulnerable [10], yet because of limited available data [11], they are rarely considered when prioritizing places for protection [12–14].

As a subsector of the global capture fisheries, SSF accounts for 90% of the world’s fishers [10] and 25% of the all catches in the sector [15]. Millions of low-income households in coastal and rural communities rely on these fisheries for their livelihoods, and the revenue generated contributes to local economies [10]. Moreover, SSF landings supply a source of protein and essential micronutrients for billions of consumers around the world [10,16,17], and play a vital role in alleviating hunger and malnutrition in developing countries [17], where more than half of all SSF catches are consumed directly by households and communities [10]. In sum, these fisheries provide important contributions to food security and poverty alleviation, especially in the developing world [10,16–18].

While definitions and terminology vary by geographic region [19], SSF can be broadly characterized as a dynamic, diverse, and complex subsector of the capture fisheries that encompasses a variety of gear types, fishing methods, and target species; along with a wide range of activities involving both men and women in the value chain for fish and fishery products [10]. Because of this diversity and complexity, data on SSF are often missing, and many fisheries remain poorly documented, especially in developing nations [11,15,20]. Based on the limited available data and the current scientific knowledge on SSF, fisheries resources in many regions appear to be declining and many may be at risk of collapse due to multiple interacting stressors, including overharvesting, environmental degradation, high indices of poverty, rapid population growth, lack of alternative livelihood options, and competition for resources [21–29].

Establishing MPAs is often advocated as an effective management tool to mitigate fishing pressures and sustain fisheries resources, in addition to conserving biodiversity [2,30]. While MPAs can support the recovery of overharvested populations and enhance fisheries productivity through the spillover effect [30–34], their initial implementation can harm fishers (e.g., food insecurity and loss of revenue) by limiting or ending access to marine resources [7,35,36]. Furthermore, issues with distribution equity–an aspect of social equity that concerns the distribution of costs and benefits [37–40]–can easily arise when negative impacts from spatial restrictions affect fishers and communities disproportionately [7,36,41,42]. In addition to the moral argument for equity [37,43], a failure to address equity concerns of MPAs may lead to conflict, reduced local support towards MPAs, and non-compliance of rules; all of which can result in suboptimal conservation outcomes [36,44,45]. While distributive concerns may be an issue within and across different stakeholder groups (e.g., communities, governments, and global stakeholders) [37], here we focus on local stakeholders who depend on SSF.

To ensure that biodiversity is represented in MPAs, and impacts on people are kept to a minimum, spatial conservation prioritization (hereafter “prioritization”) has emerged as a systematic approach for designing protected area networks [46–48]. Embedded within the broader framework of systematic conservation planning [47,48], prioritization often uses decision-support tools, integrated with spatial data on biodiversity (e.g., habitats, species distributions) and socioeconomic factors (e.g., opportunity costs such as fishing areas), to identify protected areas that achieve spatially explicit biodiversity targets with minimal impacts to marine users [46,48]. Typically, multiple data layers are included for biodiversity, whereas socioeconomic factors are reduced to one cost layer [14]. However, the release of Marxan with Zones [49], an extension of the Marxan software (the most widely used decision-support tool for conservation planning globally) [50], has provided a means to incorporate multiple socioeconomic factors in prioritization [51].

Despite recent advancements in decision-support tools, the scarcity of SSF data continues to imped efforts to represent the needs, interests, and priorities of fisheries-dependant stakeholders in prioritization [14,52]. In the absence of empirical data, it is common practice to use existing secondary data as surrogates for impacts to fisheries [14], such as density of fishing vessels [53], marine tenure [54], and population pressure [14]. The use of untested surrogates is problematic, however, because it adds uncertainties in planning processes that can compromise the integrity of MPA plans and lead to unforeseen consequences, including equity issues [14,55,56].

Previous studies have also demonstrated how the type of socioeconomic data and quality of data inputs can significantly affect the results generated in prioritization [56,57]. We focus here on two factors of socioeconomic data that may influence prioritization outcomes: data resolution (the amount of detail in the representation of features) and data completeness (a measure of the totality of features). Since commonly available socioeconomic datasets (e.g., government censuses and demographic surveys) are frequently collected at different administration levels, we define socioeconomic features represented at a local level (individual, household, or community) as “fine-resolution data” and features represented at a regional level (municipality, province, region, or country) as “coarse-resolution data”. For completeness, we use the term “complete data” to refer to a dataset with no missing socioeconomic features of interest, whereas a dataset with missing features is termed as “incomplete data”.

Collecting complete datasets on the spatial distribution of fishing activities at resolutions relevant to MPA planning will inevitably help to improve the representation of SSF in prioritization. However, efforts to address data gaps in developing countries are constrained by limited resources and low governance capacity [10,24]. In these settings, cost-effective methods are needed to collect spatial data on SFF for MPA planning. Participatory mapping with fishers–a methodology that involves the documentation and translation of fishers’ knowledge into spatial information–holds promise because fishers often have a wealth of knowledge regarding local fishing activities [58–60]. This methodology often provides an efficient and effective means of collecting spatial data on SSF in data-poor contexts [59,61–65]. The spatial data gathered can subsequently be integrated within decision-support tools to facilitate MPA planning in support of fisheries-dependant stakeholders and communities [66–68]. As a participatory approach, the methodology also emphasizes community engagement and empowerment [69–71], both of which can positively influence conservation outcomes [72–75].

The purpose of our study was to provide guidance for collecting, representing, and integrating SSF data in prioritization. We report a case study in the Philippines as a demonstration for combining SSF and biodiversity data in prioritization to design a spatially-efficient and ecologically representative MPA network with minimal and equitable impacts for SSF. Our research has two objectives. The first is to document a participatory mapping approach that recognizes and utilizes the local knowledge of fishers to collect data on the distribution of fishing activities for prioritization purposes. The second is to examine how the completeness and resolution of SSF data can influence prioritization results in terms of biodiversity representation, spatial efficiency, and distribution equity. Our prioritization results are not intended to suggest a MPA network for implementation, but to provide guidance for future prioritization applications seeking to develop MPA networks that can simultaneously achieve biodiversity objectives, promote distribution equity, and maintain SSF benefits.

## Background

The Philippines is one of six countries in the Coral Triangle, an epicenter for marine biodiversity and a global priority for conservation [76,77]. The Philippines is an interesting case study because of its high biodiversity and its many and diverse small-scale fisheries [78,79]. Coastal ecosystems and fisheries in the country are in an increasingly declining state due to several threats including overfishing, illegal and destructive fishing practices, coastal development, pollution, and climate change [80–84].

MPAs are a primary management tool for conserving biodiversity and safeguarding fisheries in the Philippines [77,85,86]. Due to the country’s decentralized resource governance system, MPAs in the Philippines have mainly been established at a community or “barangay” level (a barangay is analogous to a village community and represents the smallest political unit in the Philippines) in collaboration with municipal governments, and often with the assistance of support institutions (e.g., foreign donors, academics). Most MPAs have been planned and implemented in an ad-hoc manner, where sites have been selected based on management feasibility or local preferences, rather than biological importance [77,87]. The majority of MPAs are less than 1 km^2^ in size [85], ineffectively managed [88], and under representative of critical habitats [77,85] and key biodiversity areas (areas with the presence of globally threatened and geographically concentrated species) [89].

The Philippines is a signatory of the Coral Triangle Initiative and the Convention on Biological Diversity [3,80], and the national government has committed to protect 20% of each major marine and coastal habitat type (e.g., coral reefs, seagrass beds, mangrove forests) in no-take MPA by 2020 [80]. Furthermore, the Philippines has endorsed conservation strategies that prioritize key biodiversity areas; adopt methods and tools based on solid science and data; include and engage stakeholders; and consider equity and socioeconomic factors with an emphasis on poor and marginal communities, especially small-scale fishers and fishing communities who live in poverty, lack alternative livelihood options, and depend heavily on SSF for food and income [80,90].

Small-scale fishers in the Philippines are diverse, and nationally defined as “municipal fisherfolk” [79]. As documented in the Fisheries Code of 1998 (Republic Act 8550), a municipal fisherfolk is any person who directly or indirectly engages in fishing within municipal waters (marine tenure boundary that extends 15 km from the coastline of each coastal municipality) using a vessel up to 3 gross tons or fishing without the use of a vessel (e.g., gleaning, diving, spearfishing). In this study, we refer to these fishers simply as “small-scale fishers” and restrict our definition to people directly involved in catching fish or invertebrates in municipal waters. This definition recognizes that SSF include both men and women, a variety of targeted species, and several fishing methods that may or may not involve the use of gear or boats [91]. We do not consider commercial fisheries (fishing utilizing a vessel greater that 3 gross tons), which are prohibited from operating within municipal waters. We acknowledge that illegal commercial fishing in municipal waters remains largely unenforced and posses a serious threat to SSF [79], but this issue is beyond the scope of our research.

While the marine tenure systems in the Philippines grants small-scale fishers exclusive rights to use municipal waters in their municipality [77,87], cost restrictions (e.g., cost of boats, engines, and fuel) confine many poor fishers to operate close to home, including women who contribute to food and nutrient security of coastal households by gleaning in intertidal areas [45,91]. Given that no-take MPAs tend to be established close to shore and communities to facilitate enforcement [86,87], fishers living in communities near MPA are often displaced from their fishing ground [7,79].

The displacement of fishing effort from large MPAs can be particularly damaging when poor fishers need to invest time and resources (e.g., fuel cost) to reach further fishing grounds [92]. Hence, large MPAs are impractical and socially infeasible in this region of the world, despite being ecologically optimal [79,93]. Based on the social, economic, and governance context of the Philippines, a more realistic approach to meeting conservation commitments is to increase the number of smaller MPAs to form representative networks that distribute costs and benefits more equitably across communities [86,93,94].

## Methods

### Study area

**Fig 1:**
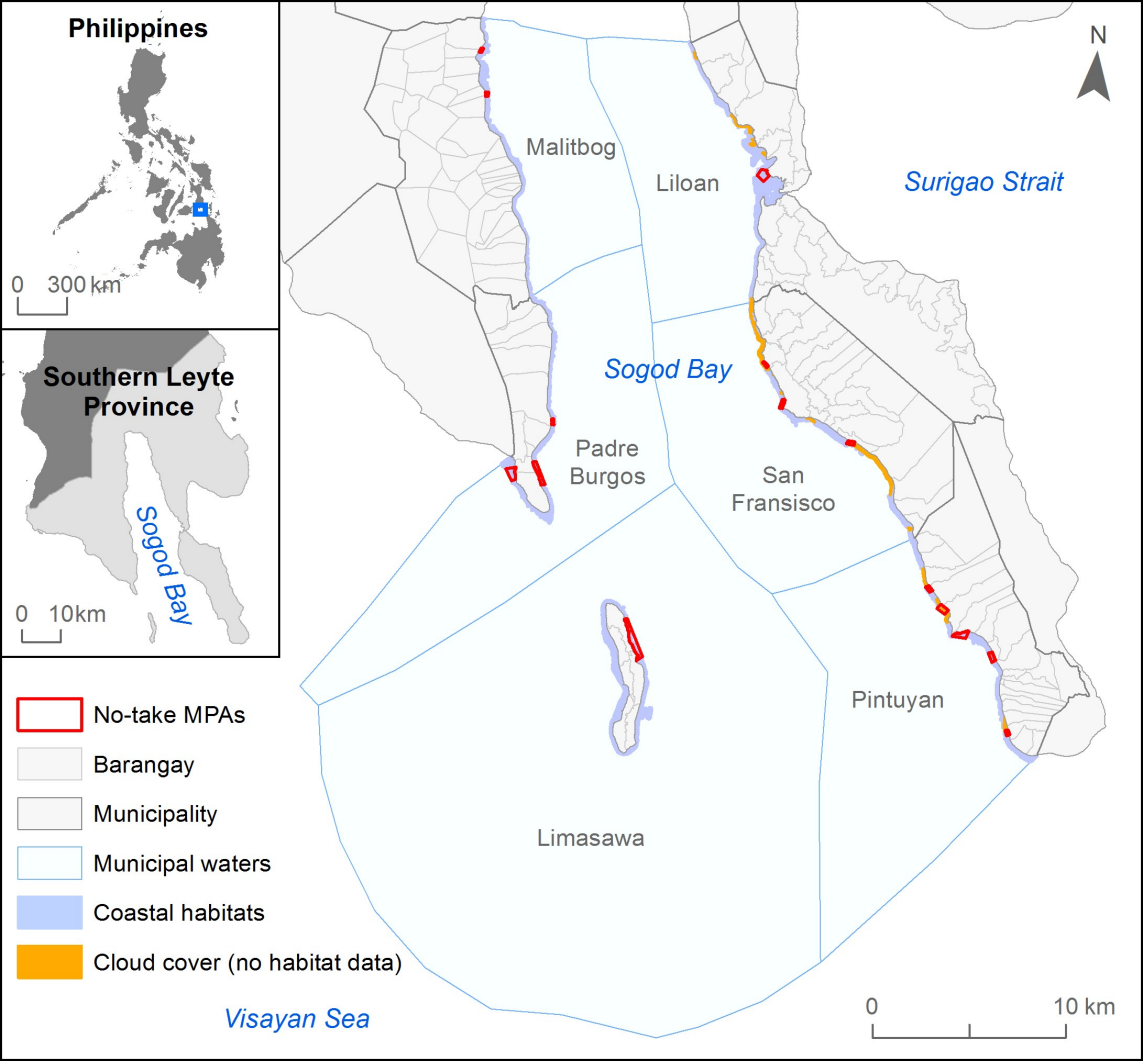
Map of the planning region in Sogod Bay, southern leyte, philippines. The planning region encompasses the municipal waters of six municipalities. The map shows the existing MPAs in the Bay and the extent of coastal habitats derived from remote sensing imagery, including gaps in data due to cloud cover.

Our case study focuses on the southern portion of Sogod Bay (9° 59.442'N, 125° 7.341'E) in Southern Leyte province, situated in the Eastern Visayan region of the Philippines (Fig 1). The planning region is defined by the municipal waters of six municipalities, which include 79 coastal communities or “barangays”. We selected the region because: (1) it is nationally recognized as a marine key biodiversity area [89] and contains a diverse range of coastal habitats including coral reefs, mangroves, and seagrass beds [95]; (2) communities have similar demographic characteristics (e.g., predominantly rural) and are highly reliant on SSF as a main source of revenue; (3) several government officials, non-government organizations, local academics, and communities support initiatives to establish new MPAs for conservation and fisheries management purposes [95]; and (4) the 16 existing MPAs reflect most MPAs in the Philippines [85,86] as they are all small (0.03 to 0.52 km^2^), individually established and managed at a community level, and collectively under representative of coastal habitats [95].

#### Fishery data

Spatial data on fisheries did not exist in the study region, and therefore we developed and held participatory mapping workshops to collect spatial data on the distribution patterns of different communities and types of fisheries (fisheries defined based on employed fishing method). A total of 779 fishers (589 men and 194 women) from the 79 coastal communities and six municipalities participated in these workshops between June 2015 and April 2016.

Each mapping workshop (one workshop per community) included six to twelve small-scale fishers from a given community. Fisher representatives (both men and women over 18 years of age) were nominated by barangay government members (e.g., the elected barangay captain or the Fisheries and Aquatic Resources Management Council) as individuals with extensive knowledge on the fishing practices in their community. To obtain a reasonably representative sample of fishers per community (it was not possible to include more 12 fisher representatives per community due to methodological challenges and time and cost restraints), we asked the government members to consider diverse sub-groups (e.g., age, gender) and types of fishers (fishers using different fishing methods) in their nominations. Participation was voluntary (no compensation provided) and identities confidential (no names recorded). We chose to obtain informed consent verbally from workshop participants as an audio recording because some fishers were illiterate. We also obtained written consent from municipal mayors and barangay captains, prior to each workshop. The informed consent, recruitment process, and mapping methodology were approved by the Human Research Ethics Board at the University of Victoria.

We developed the mapping procedures in consultation with local experts (local government offices, NGOs, academics, and fisherfolk organizations) and tested them prior to the field season via a series of “practice” workshops with volunteer fishers from communities outside of the planning region and with the assistance of Ocean-Action Resource Center, a local NGO with extensive experience working with fishing communities. This was instrumental in developing and piloting the mapping procedures and it also facilitated decisions on how to group and map fishing activities. Through these consultations and field tests, we identified 24 distinct fishing methods, which we defined based on the gear type, main catch, and generalized spatial use patterns (near-shore, far-shore, intertidal).

We conducted the mapping workshops in the Filipino dialect of Visayan. Workshops were facilitated by the lead researcher or the in-country research coordinator with the assistance of a research assistant (recorded attribute data), a local translators/assistance (acted as both a translator and mapping facilitator), a geographic information system (GIS) technician (digitized mapped data), and a data recorder (documented local knowledge and attribute data of mapped features), all of whom were trained in all procedures and protocols for mapping and recording data. The workshops generally lasted between three to six hours per community.

In each workshop, we gave participants paper maps and access to a digital map of Google Earth Pro displayed on a 20-inch touch-screen tablet. The paper maps displayed Google Earth Pro images (scale of 1:20,000) and were laminated to allow fishers to draw directly onto the maps with markers. The touch-screen tablet acted as a digital mapping tool to assist fishers to measure distances from shore and describe mapped features with greater accuracy (e.g., zoom in and out to show fishing area extent). Additionally, the tablet was connected to a keyboard and a mouse to allow the GIS technician to digitize local knowledge in situ using Google Earth Pro. The facilitator explained the scale, direction, and features (e.g., landmarks, shoreline, and islands) of the paper and digital map to minimize map bias and facilitate mapping (many fishers had a limited understanding of maps). We tested the comprehension of participants by asking them to locate certain map features.

After the mapping orientation, the research team collected information on the fisheries profile of the community (e.g., total number of motor and non-motorized boats, total number of women and men fishers, alternative sources of livelihoods) based on the local knowledge of participants. We chose to collect baseline information in this way because secondary data from local censuses were incomplete and inconsistent across communities and municipalities. For instance, fisher counts varied in whether or not they included women, minority fishing groups, and unregistered fishers (many fishers do not register with their municipality to avoid registration fees).

The collection of baseline information was followed by mapping fishing sites per method. For each fishing method, we first read the definition of the method and asked whether it had been used by any members of their community within the last 12 months. If so, we asked the group to provide general information on the seasonality, main catch, mode of transport, distances from shore, depth, and number of fishers in their community who engage in the fishing method. The participants then worked in groups of two or three to map the fishing sites of the method on a paper map. They drew the fishing sites as closed polygons within the respective municipal waters extent. We asked participants to only map sites used by fishers from the community within the past 12 months, regardless of its frequency or intensity. We asked them to exclude areas used only for transit.

We compared the drawn maps and discussed them in a group to produce a final map. The maps were often similar between participants, and small variations were addressed through group discussions. In rare cases where fishers did not agree, we chose to include all mapped fishing areas in the final map even if they differed between drawn maps. The GIS technician digitized the final map of each method based on participants’ drawings, descriptions, and distance references (e.g., distance from shore and administrative boundaries). We did this on site to allow fishers to verify the mapped fishing sites before moving on to the next fishing method. We mapped all sites within municipal waters in correspondence to the Philippines Fisheries Code. We also documented additional attribute information, such as importance value and number of fishers, and linked it to spatial data but did not use it in this study.

The spatial information obtained through the participatory mapping workshops were compiled in a GIS database in ArcGIS 10.2. The spatial data were used to examine the spatial patterns of small-scale fishers by community and fishing method, and to develop fishery features for spatial prioritization. We used the information provided by fishers to estimate the total number of fishers in each municipality and in the planning region, and to create SSF profile per fishing method.

#### Biodiversity data

Available data from benthic habitat surveys in Sogod Bay were too sparse to be useful for prioritization. Consequently, we used remote sensing to produce the first high-resolution (2 m pixel resolution) benthic habitat map of coastal areas (less than 25 m deep) in Sogod Bay. We collected data on benthic habitats using WorldView-2 satellite imagery in combination with field surveys (GPS-referenced video transects). Following standard methods used in nearshore habitats detection [96], the remote sensing analysis derived seven benthic habitat classes: rock/pebble/gravel, rocky reef, coral reef (high cover), coral reef (low cover), macroalgae, sand, and seagrass. We were unable to include deep water marine habitats in waters deeper than 25 m depth because of the limitation in using satellite remote sensing for substrate detection in deep waters [97]. The user accuracy of identification of benthic habitat classes ranged from 20% to 90%. Low accuracy rating of certain benthic classes is likely due to mismatch of field and image acquisition (four-year gap between the image acquisition date and the field surveys). Other sources of error were spectral similarities between habitat classes (e.g., spectral confusion between seagrass and macroalgae), frequent co-existing and mixing of classes (e.g., coral patches mixed with sand), and limitations of field surveys (e.g., camera tilt, low number of ground-truth points for some classes).

Coastal mangrove data were also collected through secondary sources. Datasets on mangroves were provided by the Provincial Environment and Natural Resources Management Office, the Municipal Agricultural Office, and the Municipal Planning and Development Office, and validated by local experts from Southern Leyte State University. Given the governance system in the Philippines, we subdivided the full extent of the eight habitat classes (i.e., seven classes derived from remote sensing and one from secondary sources) into six municipal waters, resulting in 45 biodiversity features (not all habitat classes were present in each municipality).

#### MPA network design

We employed the Marxan with Zones software [49], an extension of Marxan [50], to select new MPAs. Marxan with Zones employs an algorithm to produce multiple zoning options that meet spatially explicit targets based on a set of constraints and costs [49]. As initially described by Klein et al. [51], the added functionality of the tool can also be used to treat socioeconomic factors as separate features (rather than a single cost function like in Marxan) with assigned targets. Using this method, we applied MarZone to produce spatially-efficient solutions that addressed biodiversity and fishery targets simultaneously. As demonstrated in previous studies [e.g., 51,98], this method of incorporating fisheries data as ‘features’ rather than a ‘cost’ can (1) reduce the potential for conflict by facilitating planning for both biodiversity and fisheries objectives, and (2) promote the development of more socially equitable plans by distributing impacts more fairly across different fisheries features considered in the analysis.

Prior to running Marxan with Zones, we divided the planning region into 0.04 km^2^ hexagon cells or “planning units”. The size of one planning unit is comparable to the smallest no-take MPA in Southern Leyte province. Thus, MPAs selected in one or a few adjoining planning units would reflect a MPA size that could be implemented realistically in the planning region. We set Marxan with Zones to assign each planning unit in the planning region as either a no-take MPA zone or an open zone available for fishing.

In line with the Philippines national target, we set a biodiversity target to contain at least 20% of each biodiversity feature (i.e., habitat class per municipality) in the MPA zone. We assumed that conserving samples of each major habitat type across each municipality would also conserve a wide range of species, life stages, and ecosystem functions and processes. We accounted for existing MPAs by locking them into the MPA zone. To minimise the spatial restrictions of MPAs on fishery features, we set the fishery target to maintain a minimal proportion of each fishery feature in the open zone. We calculated the amount of each biodiversity and fishery feature contained in each planning unit using QGIS 2.18. We defined the cost associated with the MPA zone as the total area of planning units, based on the assumption that spatially efficient solutions are easier to implement. There was no cost for the open zone.

We developed three MPA planning scenarios to investigate how the completeness and resolution of SSF data may affect prioritization results (Table 1). All scenarios contained the same biodiversity features but fishery features varied based on two factors: data completeness and data resolution. A complete dataset included all near-shore fishing methods and an incomplete dataset only included the most dominant near-shore fishing methods. Hence, the scenarios differed in the number of fisheries considered. We excluded fishing methods used exclusively offshore in each scenario, because these methods would not be impacted by coastal MPAs (i.e., coastal habitats were targeted for MPA inclusion, therefore MPAs could only be selected in coastal areas). The fishery features in each planning scenario also varied in terms of the resolution for representing the spatial patterns associated with different fishing methods. A fine-resolution dataset represented fishing patterns at a community level (each feature corresponded to a method and a community), whereas a coarse-resolution dataset represented them at a municipal level (each feature corresponded to a method and a municipality).

**Table 1:** Fishery features in each MPA planning scenario.

Scenario	Fishery features			Type of dataset	
	N° of fishing methods ^a^	Administrative level ^b^	Total N° of features	Data completeness	Data resolution
1	17	Community	868	Complete	Fine
2	5 (Most dominant methods)	Community	362	Incomplete	Fine
3	17	Municipal	145	Complete	Coarse

^a^ Does not include fishing methods used exclusively off-shore.

^b^ Fishing methods were grouped by barangay/community or municipality.

**Scenario one** represented a complete and fine-resolution SSF dataset, because it incorporated SSF that included all fishing methods at a resolution of communities. In comparison, **scenario two** only included the five most used fishing methods at risk by coastal MPAs at a resolution of communities, thereby representing an incomplete and fine-resolution dataset. We compared scenario one and two to analyse the benefits and trade-offs of using complete versus incomplete datasets on SSF. **Scenario three** incorporated all fishing methods at a resolution of municipalities (fishing methods grouped by municipality), where spatial data on community fishing grounds were merged by municipality. It represented a complete and coarse-resolution dataset and was compared to scenario one to examine the impacts of using fine-resolution fishery data linked to community as opposed to coarse-scale resolution data that aggregates data at a municipal level.

We first ran scenario one to identify the maximum fishery target that could be achieved while still meeting the biodiversity target. This was done by running Marxan with Zones iteratively with a constant biodiversity target and with a series of increasing fishery targets that varied by 1% increments. With each series, we calibrated the feature penalty factor (fpf) by increasing it until all biodiversity features met the biodiversity target (same fpf for each biodiversity feature). We then increased the fpf of each fishery feature (same for each fishery feature) until all fishery targets were achieved. We gave preference to achieving the fishery target by allowing biodiversity features to suffer the shortfall when both the biodiversity and fishery targets could not be met simultaneously. Running the calibration with a series of increasing fishery targets allowed us to identify the highest fishery target that could be achieved (all fishery features met targets) while simultaneously meeting all biodiversity targets. For scenario one, targets for each biodiversity and fishery features could be met up to a maximum fishery target of 75%. We then applied this target to run scenarios two and three, along with the above fpf calibration technique.

Each scenario was set to produce 100 solutions or MPA plans with 100 million iterations. We utilised the “best solution” and the “selection frequency” outputs of each MarZone analysis to analyse the scenarios. The “best solution” was used to identify the most efficient MPA plan (based on the lowest scoring of 100 output solutions), whereas the “selection frequency” illustrated the number of times each planning unit was selected in the MPA zone from all runs in a scenario.

We analysed the “best solution” of each scenario to determine the proportion of biodiversity and fishery features that achieved their targets. In all cases, we evaluated distribution equity based on predicted impacts on opportunity costs (loss of fishing grounds). Specifically, distribution equity was assessed as a measure of the distribution of fishing area lost in MPAs among all fishery features at a resolution of communities (features itemized by fishing method and community; included all methods that could be impacted by coastal MPAs), calculating the proportion of each fishery feature lost in the MPA zone under each scenario. We measured spatial efficiency by the total area of planning units in the MPA zones of the 100 solutions derived from each scenario.

## Results

### Fisheries profile

The spatial use patterns of small-scale fishers varied by community and fishing method. For instance, gleaning (hand collection of prey in intertidal areas) and trap fishing (e.g., squid and fish traps) were typically practiced close to shore, whereas multiple handline and certain forms of squid fishing were exclusively used offshore (i.e., they did not overlap with coastal habitats).

**Fig 2:**
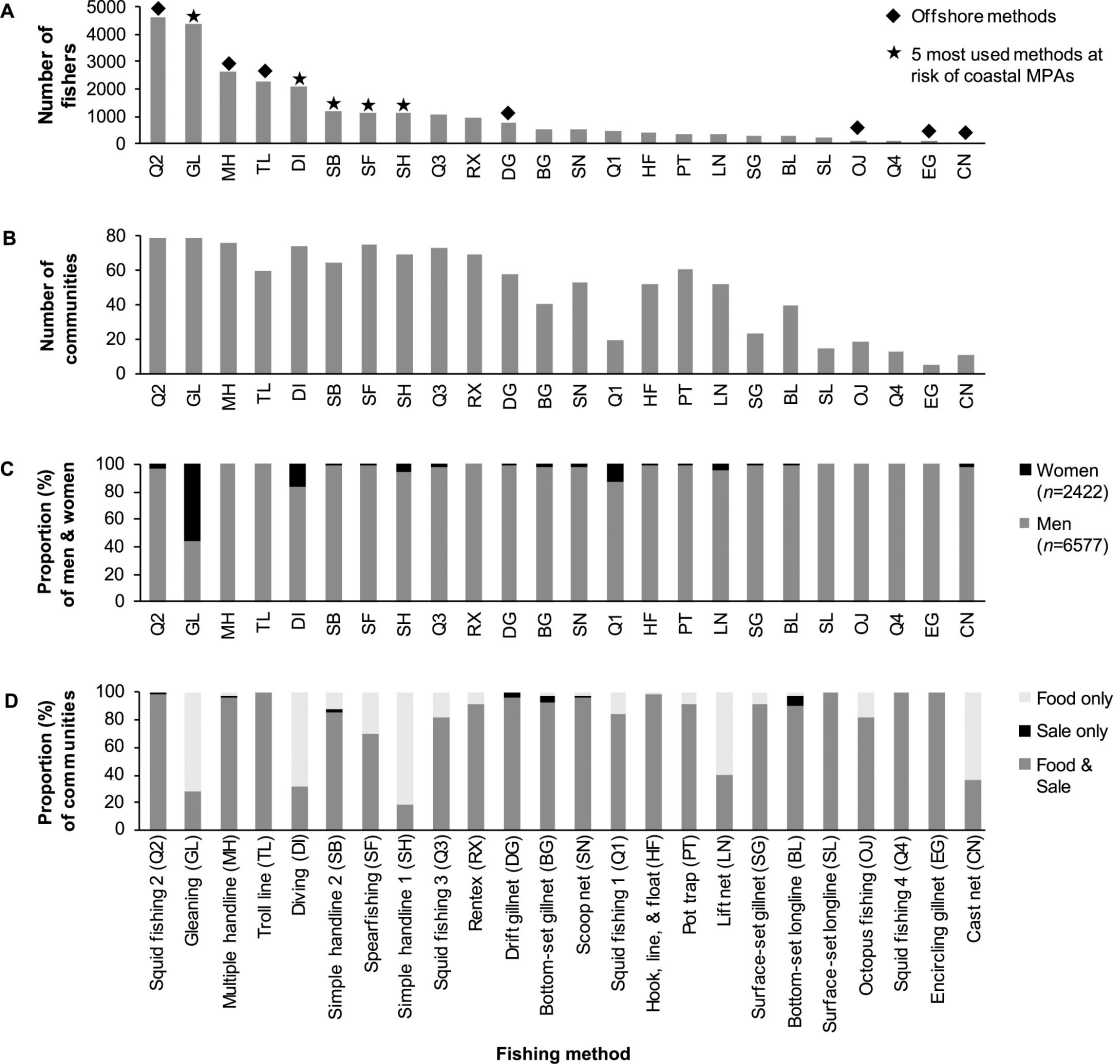
Small-scale fisheries profile by fishing method. (A) The total number of fishers that actively participate in fishing, by method. (B) The total number of communities with fishers that employ a given fishing method, by method. (C) The proportional participation of men and women per method. (D) The proportional distribution of communities that fish for food, sale, or both food and sale, by method.

Based on the local knowledge of fisher participants, we estimated that there are approximately 9000 small-scale fishers (6577 men and 2422 women) in the planning region (average of 114 fishers per community, range of 15 to 650 fishers per community). Fishers often employ more than one form of fishing, so the sum of fishers per method shown in Fig 2A does not equate to the total number of fishers in the planning region. Excluding fishing methods used exclusively offshore (Fig 2A), the following are the five most used methods at risk of coastal MPA: gleaning, diving, spearfishing, simple handline 2 (handline with a vessel), and simple handline 1 (handline from shore). These methods were practiced by a high proportion (81 to 100%) of coastal communities (Fig 2B), although the methods used per community did vary.

The number and proportion of fishers per method varied by community and municipality. All forms of fishing were practiced and dominated by male fishers, with the exception of gleaning (Fig 2C). Most women fishers participated in gleaning, which was described by many participants as an important source of food for their household and community. A small portion of women also participated in near-shore fishing methods (e.g., diving and simple handline), often in the company of male relatives. Most fishing methods were used to obtain food and income (Fig 2D), although a few communities employed certain methods just for food (e.g., gleaning, diving) or sale (e.g., bottom-set longline, drift gillnet).

### Biodiversity representation

We identified the “best” solution and the “selection frequency” of each MPA planning scenario, which varied spatially. The best MPA plan of each scenario ensured that a minimum of 20% of each habitat class per municipality was conserved in MPAs (Fig 4A, 4B and 4C). Hence, all scenarios were capable of achieving the same biodiversity representation target.

### Distribution equity

**Fig 3:**
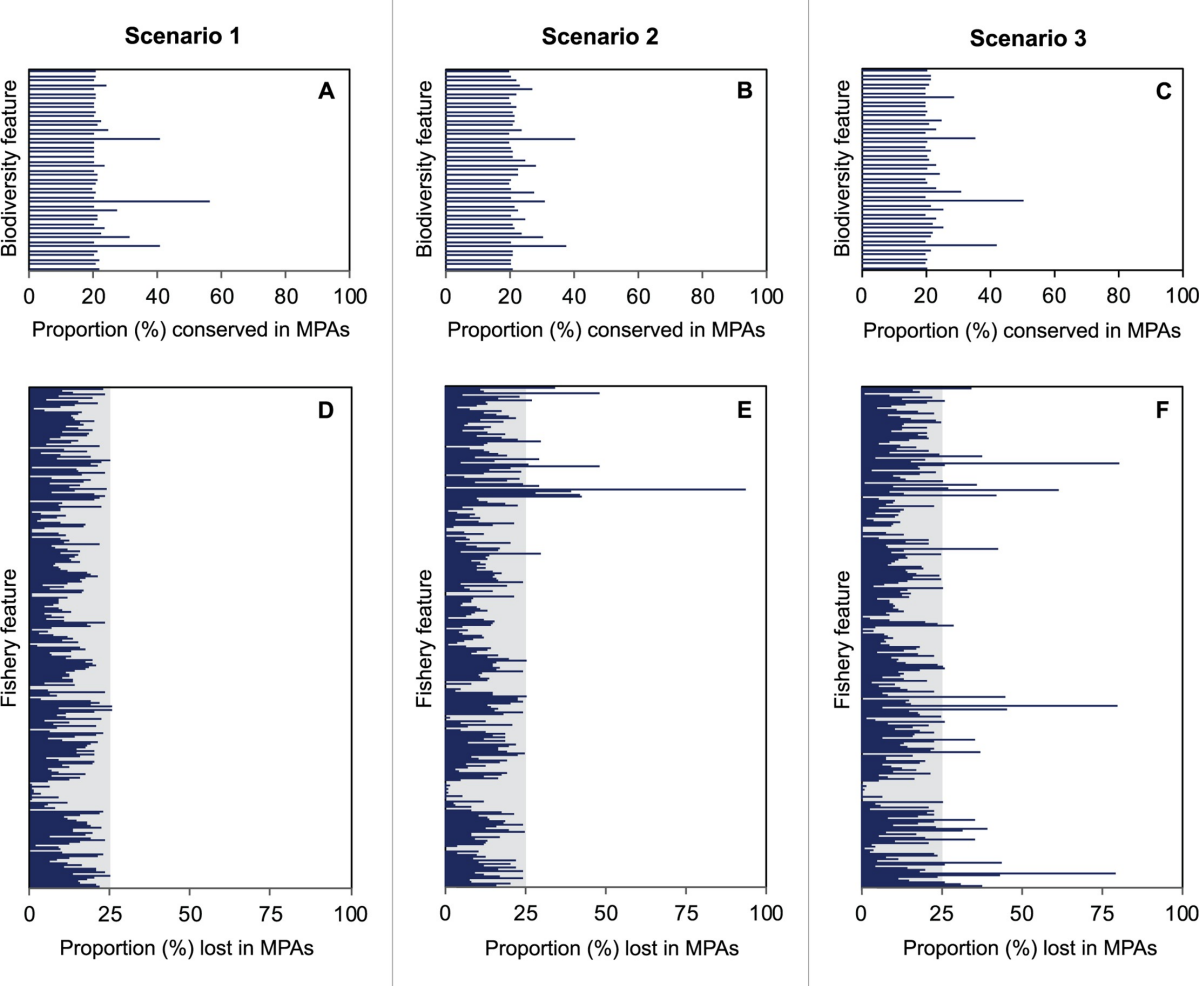
The proportion of biodiversity and fishery features in MPAs by scenario. The results are based on MPAs selected in the “best” MPA network plan of each scenario. Each bar in the top graphs (A-C) represent biodiversity features (coastal habitat class per municipality) conserved in MPAs. In all cases, the biodiversity features met their 20% representation target for inclusion in MPAs. Each bar in the bottom graphs (D-F) represent a fishery feature at a resolution of communities (fishing method per community) and shows the proportion of each feature that would be lost in MPAs. Fishery features contained within the shaded zone of each graph have maintained at least 75% of their fishing area in the open zone (i.e., they have lost less than 25% of their entire fishing area in new MPAs).

While each scenario met the fishery target successfully, they had different fishery features. Comparisons of the best MPA plan of each scenario in terms of their impacts on all fisheries at a resolution of communities (i.e., 863 features; each defined by method and barangay), showed that scenario one was the best at maintaining distribution equity (Fig 3D), followed by scenario two (Fig 3E) and three (Fig 3F).

**Fig 4:**
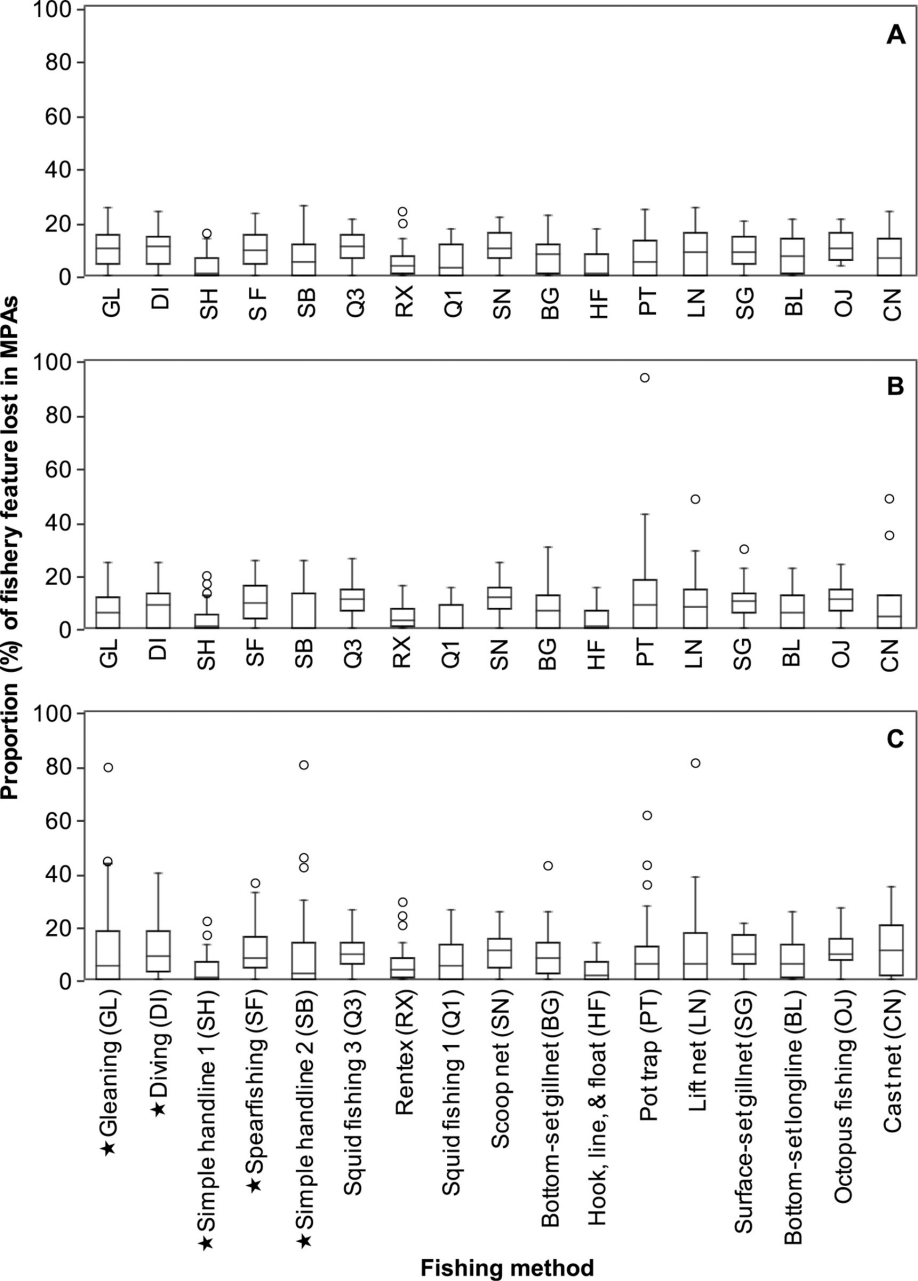
The proportion of each fishery feature lost in MPAs by scenario. The results are based on the “best” MPA network plan derived from (A) scenario one, (B) scenario two, and (C) scenario three. The fishery features are expressed at a resolution of communities (method per community), and dominant fishing methods are annotated with a “⋆”. Each boxplot shows the range, upper and lower quartiles, and the median values of fishery features by method. Outliers are shown as open circles.

Scenario one was the only scenario that considered all communities and types of fishers in the Marxan with Zones analysis. In doing so, it resulted in a MPA plan that distributed costs equitably across all fishery features at a resolution of communities, ensuring that all communities maintained at least 75% of the fishing area of each method (Fig 4A). In comparison, the best MPA plan from scenario two raised equity concerns as it only considered the most popular methods in the planning region at a resolution of communities. Still, 98% of fishery features (8 of 863 features), including many of those associated with less popular methods (not considered explicitly in scenario two), maintained a minimum of 75% of their fishing areas (Fig 4B). The remaining 2% were associated with less popular methods that lost more than 25% of their total fishing area. This included one community that would lose 93% of their pot trap fishing sites.

In comparison to scenarios one and two, we found that grouping fishers by methods and municipality in scenario three resulted in the most inequitable distributions of MPA costs for fisheries associated with different fishing methods and communities. The best MPA plan of scenario three caused 5% of fishery features at a community scale (42 out of 863) to lose more than 25% of their fishing grounds, including four features that suffered losses between 61 to 80% (Fig 4C).

### Spatial efficiency

**Fig 5:**
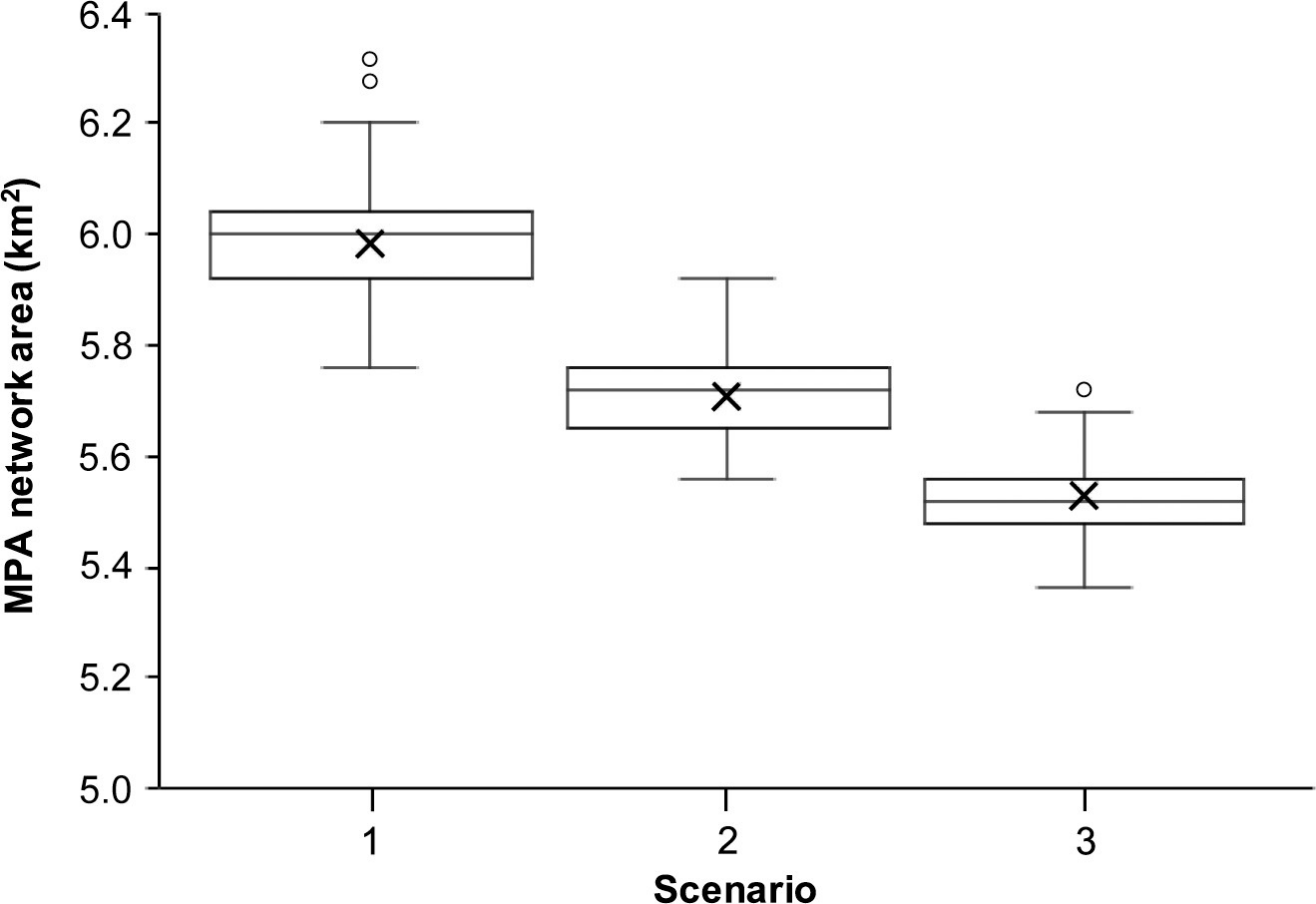
The total area of planning units contained in the MPA zone under different scenarios. Boxplots show the range, upper and lower quartiles, and median values of 100 replicate Marxan with Zones solutions. The mean is shown by an “x”.

The average total area of MPA planning units selected in the MPA zone of 100 replicate Marxan with Zones solutions was highest for scenario one, followed by scenario two and three (Fig 5). Including all fishing methods at a resolution of communities (scenario one) resulted in a total MPA zone area that was on average 4.7% larger than the scenario that included only the most used fishing methods at a community scale (scenario two), and 7.6% larger than the scenario that considered all fishing methods at a resolution of municipalities (scenario three).

## Discussion

The inclusion of socioeconomic and equity considerations in MPA planning is widely recognized as fundamental to biodiversity conservation [99–102], and increasingly addressed in prioritization applications [103–108]. In practice, however, such considerations have rarely extended to SSF, largely due to data limitations [see exceptions 63,67]. Here, we illustrate the value of employing participatory mapping with local fishers to collect spatial data on SSF for prioritization, specifically in the context of designing a MPA networks in a data-poor region with high dependence on coastal resources. Using different planning scenarios, we also highlight the benefits of including complete and fine-resolution data on SSF in prioritization in terms of promoting distribution equity, and points to the potential pitfalls of oversimplifying these fisheries.

We focused on the collection of SSF data through participatory mapping, a methodology that is increasingly used to address data gaps on fisheries in prioritization assessments [58,61,62,106,109]. Employing this methodology in our study facilitated the prioritization process in two major ways. First, it allowed us to develop a greater understanding of the diversity and characteristics of SSF in the planning region, which was useful for informing decisions for the inclusion of SSF data in prioritization. Second, it provided an efficient and effective data-collection method that successfully captures the spatial variations in resources use patterns of different fisheries and communities in the planning region. This is a major step forward from many prioritization assessments that have relied on incomplete data and untested surrogates that are less likely to reflect how fishers use coastal and marine resources [e.g., 51,54,98,103]. Moreover, the inclusion of fishers’ knowledge in conservation planning presents a broader view of expertise and knowledge sources in comparison to conventional conservation approaches that have predominantly relied on “expert” opinions and scientifically-derived knowledge [59,110].

As observed in similar studies [63,67,109], fishing communities and stakeholders in Sogod Bay were willing to share their local knowledge on fishing activities. Through this knowledge source, we found that SSF in the planning region had many common characteristics of SSF studied in other geographic regions [26,91,111–113]. They encompassed multiple fishing methods (mainly methods using low technology or less advanced gear), which were either used for home consumption or sold in local markets within or close to their communities. Most forms of fishing were closely tied to coastal communities, as many near-shore fishing methods were practiced in areas close to the communities were fishers live. Many fishers also described using fishing methods that have historically been practiced in their community. Like other places in the Philippines [91], women who have rarely been included in fisher counts represented a substantial portion of the all fishers in Sogod Bay, especially amongst those engaged in gleaning. Based on these characteristics, SSF data used in prioritization should reflect different uses, genders, and forms of fishing to avoid marginalizing fishers and their fisheries in MPA planning.

As demonstrated through the results of the planning scenarios, prioritization assessment that include complete and fine-resolution data input on SSF, reflecting variations in the spatial use patterns of different fisheries and communities, can derive solutions that are less detrimental and more equitable across all fisheries and communities, without compromising important conservation objectives like biodiversity representation. Additionally, the scenarios illustrate how the results depicting estimated impacts on different stakeholder groups and communities are dependent on how stakeholders are defined and grouped, and how socioeconomic data are collected [114].

Opportunity costs for fisheries are a typical and predictable impact associated with the initial establishment of MPAs, and a common metric of cost of SCP assessments [14,100]. While this area-based metric ensured that a minimum proportion of each fishery feature is kept open to fishing, it is still a surrogate for distribution equity. It does not account for the behaviour, priorities, preferences, and adaptive capacity of fishers (e.g., fishers differ in their ability to switch to other forms of fishing and access new fishing grounds) [106,115,116], nor does it consider the array of social, economic, political, and institutional that may be important for determining the actual impacts of different plans on fishers and communities [9]. However, integrating multiple factors within the input parameters of a Marxan with Zones analysis is challenging and requires making subjective decisions (e.g., how to set targets based on importance value, how to calibrate different metrics and targets simultaneously) [100]. Hence, we chose a simpler area-based metric to minimize uncertainties.

To our knowledge, only a few prioritization assessments in the literature have addressed distribution equity for fisheries at a community level [63,67]. Given the diversity of methods and spatial patterns we observed amongst fishers and communities in Sogod Bay, we did expect the findings of the scenario three that incorporating fishing data linked to communities would produce more equitable plans. As discussed in Mcdermott et al. [117], social characteristics and inequities between localities or communities need to be considerate at this resolution. For scenario two that only considered dominant fisheries, we found the proportion of fishery features that incurred high costs was unexpectedly low for a process, even amongst minority fisheries that were not included in prioritization. One possible explanation is that the dominant fisheries in Sogod Bay included fishing methods (e.g., simple handline, gleaning, diving) that are typically practiced close to a fisher’s community and often restricted to narrow areas close to the coastline, which is a similar spatial pattern as some less dominant fishing methods (e.g., octopus fishing, bottom-set gillnets).

We acknowledge that it may not be feasible or even necessary to include all fisheries or communities in spatial prioritization (e.g., fishers may only be interested in maintaining important fishing grounds). As shown in our results, one possible critique of including all fisheries and communities in spatial prioritization is that it can result in less spatially efficient plans. While the total area of planning units in MPAs under the two scenarios did not differ considerably, we do acknowledge that spatially efficient plans may be easier to implement, especially in nations with centralized governance systems. However, we agree with Game et al. [109] that spatial inefficiency is probably a poor indicator of social acceptability in the context of developing countries, especially countries like the Philippines that have decentralized governance systems. Thus, we argue that spatial efficiency should not be favored over considerations of equity.

Efforts to address distributive concerns in MPA planning do not ensure that any resulting actions will be equitable or perceived to be equitable among stakeholders. Conservation practitioners need to be aware of differing stakeholder perspectives and power dynamics between stakeholder groups affecting planning and decision-making processes [101,118,119]. Furthermore, it must be made clear that MPAs designed using decision-support tools, including those in our study, are not meant to reflect the final design of a network. They require fine-tuning to consider a wide range of ecological, political, socioeconomic, and practical factors [50]. For instance, additional ecological information (e.g., species distributions, habitat quality, and bathymetry data) could supplement the remote sensing outputs and strengthen the analysis. Other important considerations include MPA design parameters (e.g., size, spacing, and connectivity) [120,121], management effectiveness [88], governance capacity [122], informed opportunities (e.g., local communities in support of establishing MPAs) [109,123], and other types of marine users (e.g., tourism sector) [124].

Additional research is required to further enhance understanding of SSF and inform better decisions on how to collect and integrate data on fisheries-dependant stakeholders and communities in prioritization, especially when considering socioeconomic factors that affect SSF which are difficult to capture using quantitative data collection methods. We contribute towards addressing this knowledge gap by presenting a cost-effective and participatory method for collecting spatial data on the distribution of SSF activities; and by providing a demonstration of how the completeness and resolution of data inputs on SSF can affect prioritization outcomes, particularly in regard to distribution equity. Our results highlight the dynamic and complex nature of SSF, along with the dangers of neglecting to reflect the heterogeneity of these fisheries in data inputs within prioritization assessments. While our focus is on prioritization, the significance of these findings extent to multiple conservation strategies, targets, and treaties; along with the upcoming post-2020 global Strategic Plan for Conservation.
